# Editorial: Biomarkers of non-motor symptoms in Parkinson's disease and parkinsonisms

**DOI:** 10.3389/fneur.2023.1257064

**Published:** 2023-09-11

**Authors:** Massimo Filippi, Roberta Balestrino, Federica Agosta

**Affiliations:** ^1^Faculty of Medicine and Surgery, Vita-Salute San Raffaele University, Milan, Lombardy, Italy; ^2^Neurology Unit, IRCCS San Raffaele Scientific Institute, Milan, Italy; ^3^Neuroimaging Research Unit, Division of Neuroscience, IRCCS San Raffaele Scientific Institute, Milan, Italy

**Keywords:** Parkinson, parkinsonism, biomarker, non-motor symptoms, neurodegeneration

The diagnosis of Parkinson's disease (PD) is clinical and relies on the presence of motor symptoms, namely parkinsonism (defined as the presence of bradykinesia and rest tremor and/or rigidity) (1). However, PD cardinal and defining motor symptoms of PD also occur in other disorders (here generally referred to as “parkinsonisms”) and the clinical phenotype, especially at the onset of the disease, can encompass more than one pathophysiological entity. PD is a progressive neurodegenerative disease: the progression of the disease is not linear and differs among patients in terms of trajectory, severity, and manifestations. The inability of capturing these differences within the entity of PD is thought to be one of the major contributors to the failure of neuroprotective trials (2).

For most patients, the late stage of the disease is characterized by motor complications and non-motor symptoms (NMS) (3). Indeed, despite being classically defined by its motor characteristic, PD is a complex disease with several NMS. NMS (encompassing olfactory and autonomic dysfunctions, psychiatric symptoms, sleep disturbances and cognitive impairment, among others) play a relevant role through all the phases of the disease—aiding the diagnostic process in early stages, being determinant for quality of life and autonomy during disease progression and being a major determinant of mortality in later stages (4). Importantly, NMS also characterize the prodromal phase, when pathological processes and neurodegeneration have begun but cardinal motor symptoms have not developed yet: the identification of this phase is critical for clinical trials testing disease-modifying treatments (5).

The accuracy of clinical diagnosis in PD and parkinsonism is far from complete and highly dependent on the experience of the clinician; moreover, the great variability of evolution in terms of severity and clinical picture further complicates the assessment of disease progression: these issues constitute a critical limitation in clinical and research practice. Currently, the gold standard for measuring disease progression is the Movement Disorder Society's Unified Parkinson's Disease Rating Scale (MDS-UPDRS): even though it has been extensively validated and it is widely used worldwide, it is a suboptimal primary outcome measure in trials of disease modification due to its limitations, e.g., intra- and inter-rater reliability issues, and susceptibility to symptomatic treatment related variability, floor effect, and only partial ability to capture the abovementioned complexity and heterogenicity of the disease (6). Indeed, it is widely recognized that reliable biomarkers are urgently needed in PD, both in clinical and research settings, with the aim to improve diagnostic accuracy—even in the prodromal phase—, disease progression tracking and patient stratification, in order to deliver personalized treatment and optimize trial design. Despite significant effort in this direction, to date this is still an unmet need.

In a classic definition, a biomarker is “a characteristic that is objectively measured and evaluated as an indicator of normal biologic processes, pathogenic processes, or pharmacologic responses to a therapeutic intervention” (7). Good biomarkers should be measurable with little or no variability, should have a sizeable signal to noise ratio, and should change promptly and reliably in response to changes in the condition or its therapy (8). Besides concerns regarding the validation of biomarkers, the feasibility and clinical utility are other aspects that should be considered.

Based on their use, biomarkers can be classified as diagnostic, monitoring, prognostic, predictive, and safety biomarkers. In the search for biomarkers, it is pivotal to know the pathophysiological relationship between the candidate biomarker and the clinical endpoint. The pathophysiology of PD—as its clinical manifestations—is complex and cannot be attributed to one mechanism of disease: several pathways, such as mitochondrial and lysosomal dysfunction, protein misfolding, oxidative stress, and inflammation/neuroinflammation (to name a few) have been implicated in its pathogenesis. Similarly, the development of specific symptoms is a multifactorial process, and it is not only explained by the degeneration of the substantia nigra. In PD, the identification of reliable biomarkers might improve the accuracy of early diagnosis, clarify subtypes, and accelerate clinical trials. In response to this unaddressed need and thanks to advances in technology, recent years have seen an exponential increase in research in the field. Several potential biomarkers (3) are currently under investigation to answer to this urgent question including clinical, digital—e.g., data obtained by gait analysis or wearable sensors—, neurophysiological—e.g., advanced EEG analysis—, imaging—such as structural and functional magnetic resonance imaging (MRI), PET and SPECT—biological/biofluid—e.g., CSF and blood biomarkers such as α-synuclein species, markers of amyloid and tau pathology, lysosomal enzymes, neurofilament light chain—, histological—e.g., biopsies of peripheral tissues such as gastrointestinal mucosae and salivary glands—, and genetic—e.g., high-penetrance mutations (e.g., *SNCA, VPS35*, biallelic *PRKN/PINK1/DJ1* etc.), intermediate penetrance mutations (e.g., *LRRK2* G2019S), low penetrance genetic risk factor variants (e.g., *GBA* variants), and other variants that have been linked with specific clinical features. However, to date, no single biomarker is univocally accepted for the diagnosis in PD, which remains clinical. The lack of sensitivity in the earliest stage of the disease or the lack of specificity in differentiating with other causes of parkinsonism are among the greater challenges in this field. The identification of a disease-progression biomarker is also a complex matter, given the variability and heterogenicity of motor or cognitive trajectories in patients with PD. For example, although dopamine transporter single-photon emission computed tomography (DaT-SPECT) can be used to assess dopaminergic denervation by detecting loss of striatal DaT already in very early stages of PD (even in the prodromal phase), it is not useful to differentiate among degenerative forms of parkinsonism, shows a weak correlation with PD severity and progression and it is subject to some pitfalls (9). A similar balance of pros and cons can be found in other candidate biomarkers for PD (10). The aim of the present Research Topic, considering the great interest surrounding the research of biomarkers in PD and considering the importance of NMS in every stage of the disease and their pivotal role in capturing the multifaceted aspects of PD, is to focus on studies that have contributed to the research in the field of biomarkers in PD focusing on NMS.

## Author contributions

MF: Conceptualization, Supervision, Writing—review and editing. RB: Writing—original draft. FA: Conceptualization, Supervision, Writing—review and editing.
